# An Image Encryption Algorithm Based on Random Hamiltonian Path

**DOI:** 10.3390/e22010073

**Published:** 2020-01-06

**Authors:** Wei Zhang, Shuwen Wang, Weijie Han, Hai Yu, Zhiliang Zhu

**Affiliations:** Software College, Northeastern University, No.11, Lane 3, Wenhua Road, Shenyang 110819, China; zhangwei@swc.neu.edu.cn (W.Z.); cococuby@163.com (W.H.); yuh@swc.neu.edu.cn (H.Y.); swc.zhuzhiliang@gmail.com (Z.Z.)

**Keywords:** image encryption, Hamiltonian path, Bernoulli map, chaotic system

## Abstract

In graph theory, Hamiltonian path refers to the path that visits each vertex exactly once. In this paper, we designed a method to generate random Hamiltonian path within digital images, which is equivalent to permutation in image encryption. By these means, building a Hamiltonian path across bit planes can shuffle the distribution of the pixel’s bits. Furthermore, a similar thought can be applied for the substitution of pixel’s grey levels. To ensure the randomness of the generated Hamiltonian path, an adjusted Bernoulli map is proposed. By adopting these novel techniques, a bit-level image encryption scheme was devised. Evaluation of simulation results proves that the proposed scheme reached fair performance. In addition, a common flaw in calculating correlation coefficients of adjacent pixels was pinpointed by us. After enhancement, correlation coefficient becomes a stricter criterion for image encryption algorithms.

## 1. Introduction

When computers and the internet came on the scene, here came the era of information, accompanied by the formidable challenge of information security. Among complicated information, the vivid multimedia information is preferred by people, especially digital images. Consequently, such information involves both collective interests and personal interests. For instance, images of military affairs are related to the safety of whole country. Privacy and copyright of images influence everyone’s peace of mind. To protect the rights of image’s owners, methods like steganography, watermarking, and encryption are frequently utilized [1]. Among these techniques, encryption is a direct and thorough means. Nowadays, image encryption is an inviting and fruitful field, and many imaginative image encryption algorithms are proposed.

One picture is worth more than ten thousand words, and there indeed are tens of thousands of pixels in a digital image. To encrypt the bulk data of images, traditional cryptosystems are not efficient enough. Among specific image encryption schemes, the permutation–diffusion structure is widely used. Essentially, permutation is to rearrange image pixels on different dimensions. In [2], 2D CMT (chaotic magic transform) was proposed for permutation. In [3], image scrambling was performed by a parametric 2D Sudoku matrix. In [4], horizontal and vertical wave lines were utilized to realize row rotation and column rotation. This is also a 2D method. In [5], spatial permutation was performed on a 3D bit matrix by using orthogonal Latin cubes. Moreover, file-based algorithms like [6] deem images as 1D binary files when realizing permutation. Considering the features of bit distribution in digital images, encryption schemes [7,8] with bit-level permutation are proposed.

Sometimes, the permutation phase is accompanied by a sort operation, such as [2]. However, time complexity of sorting is usually nonlinear. To obtain high efficiency, the additional operation should be avoided. Regarding an image as a 1D pixel array, permutation can be depicted as an arrangement of pixels, which is represented in the bijective map from plain image to permuted image. If we connect the pixels by order of the arrangement, all the pixels are traversed exactly once. Deeming pixels as the vertices of a graph, such a path of traversing is known as a Hamiltonian path in graph theory. Conversely, a Hamiltonian path corresponds to an arrangement of permutations. Following this thought, the method of building Hamiltonian path is equal to a permutation scheme. As a Hamiltonian path can be generated without sort operation, the corresponding permutation algorithm has the advantage of efficiency. In cryptography, substitution is a classical method of cipher schemes. To substitute pixel’s grey value, arrangement of all the possible grey levels is requisite. Hence, the thought of random Hamiltonian path is also suitable for the substitution of grey levels.

It is common knowledge that chaotic systems have conspicuous advantages for cryptosystems. High dimension chaotic systems possess complex chaotic behavior, while 1D chaotic systems are convenient for implementation. Under synthetical consideration, some combined chaotic maps have been explored in recent encryption schemes [2,9,10,11,12]. Just resembling the series-parallel connection of resistors in circuits, these chaotic maps are a combination or adjustment of the original chaotic maps. Multiple chaotic maps can be coupled as CML (chaotic map lattice) [13,14]. By these means, chaotic behavior is magnified, leading to better chaotic performance. In this paper, a new chaotic map was also explored.

There are some innovative works in this paper:

- A method of building a random Hamiltonian path within digital images was designed, which is equivalent to permutation. On this basis, bit-level permutation of high efficiency was achieved.
- Following the thought of the random Hamiltonian path, arrays for grey levels’ substitution can be generated.
- An adjusted Bernoulli map is proposed, which is suitable for image encryption schemes.
- The ambiguous definition of diagonal direction is normalized to two orthogonal directions when calculating correlation coefficients.

The rest of this paper is organized as following: Section 2 explains the Hamiltonian path and the procedures to generate such paths within images. Section 3 expounds the adjusted Bernoulli map. In Section 4 the proposed scheme is thoroughly introduced. The results of simulation experiments are exhibited in Section 5. Section 6 is the summary of the entire paper.

## 2. Hamiltonian Path

As was mentioned earlier, the scheme of generating a random Hamiltonian path is tantamount to permutation. In this section, the relevant theories are presented, while the method of generating a Hamiltonian path is proposed.

### 2.1. Basic Theory of Hamiltonian Path

Graph theory is a classical branch of mathematics. The term graph refers to the figures composed by points and the connecting lines between the points. Commonly, the points are called vertices, and the lines are called edges. The definition of graph is *G* = (*V*, *E*). Here *V* is a nonempty set of finite vertices and the set of edges *E* = {(*x*, *y*)|*x*, *y* ∈ *V*}. If the vertex pair (*x*, *y*) in *E* is ordered, the graph is named a direct graph. Otherwise, it is named an undirected graph.

In an undirected graph, a path *P* is a sequence of vertices *v*_1_ *v*_2_ … *v_k_*, and there exists anedge between each of the vertex pairs *v_i_ v_i_*_+1_. The *k* is the number of vertices that *P* contains, in other words, the length of *P*.

**Figure 1:**
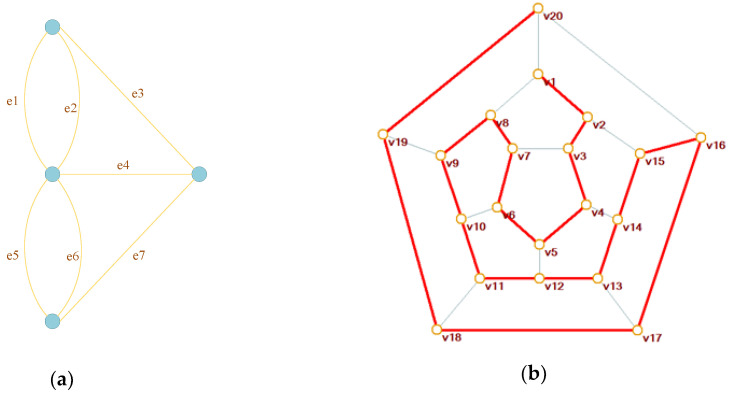
(**a**) The problem of Konigsberg bridges and (**b**) Hamiltonian path.

There are two special categories of path, Euler path and Hamiltonian path. Euler path refers to the paths that traverse each edge once and only once. A famous instance is the problem of Konigsberg bridges [15]. In 1736, Leonhard Euler had proved that there is no solution for the problem. This is known as the beginning of graph theory. Hamiltonian path refers to the paths that traverse each node once and only once, or an arrangement of all vertices in which every adjacent vertex pair is connected by at least one edge. The problem of Hamiltonian path can be traced back to 1859, when Willian Hamilton talked about a mathematical game: traverse all the vertices of a dodecahedron and pass by the vertices exactly once. Figure 1 is the graphic illustration of the two famous problems.

Graphs are generally intricate. The problem of finding a Hamiltonian path is a nondeterministic polynomial complete problem (NP-C problem), one of the most burdensome challenges in mathematics [16,17,18,19]. Off the beaten track, DNA computing [20] and light-based computers [21] have been developed to solve this problem efficiently. However, generating a Hamiltonian path within digital images can be much easier.

### 2.2. Hamiltonian Path Within Digital Images

**Figure 2:**
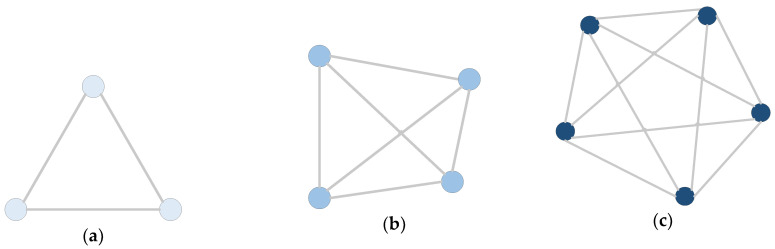
Complete graphs. (**a**) Three nodes, (**b**) Four nodes, (**c**) Five nodes.

For an undirected graph of *N* vertices, there are, at most, *N* × (*N* + 1)/2 edges. Under this condition, any two vertices are connected by an edge. Such a graph is called a complete graph. Some instances of complete graphs are shown in Figure 2. The complete graphs with three nodes, four nodes, and five nodes are shown in Figure 2a–c, respectively.

There are varieties of theorems to measure whether there are Hamiltonian paths in a graph. One of the theorems is as below:

Dirac theorem: In a graph *G* of *N* vertices, if for each vertex *v_i_* there always is *d*(*v_i_*) ≥ *N*/2, then at least one Hamiltonian path exists in *G*.

The *d*(*v_i_*), otherwise called the degree of *v_i_*, represents the quantity of edges connected with *v_i_*.

In our scheme, digital images were regarded as complete graphs. Hereof, the pixels are the vertices, and there is an edge between every two pixels. According to Dirac theorem, there always exist Hamiltonian paths in such graphs.

To build a Hamiltonian path within an image, pixels are divided into two parts. One is composed by the pixels that have been added into the path. The other one is composed by the rest of the pixels. Firstly, a pixel is chosen to be the path’s outset. Then, the other pixels are added to the path one by one. If the image’s size is *M* × *N* and its pixels are {*P*_1_, *P*_2_, …, *P_M_*_×*N*_}, this progress can be generalized as the following steps:

**Step 1:** Choose a pixel from {*P*_1_, *P*_2_, …, *P_M_*_×*N*_} and put it in the position of *P_M_*_×*N*_.

**Step 2:** Choose a pixel from {*P*_1_, *P*_2_, …, *P_M_*_×*N*-1_} and put it in the position of *P_M_*_×*N*-1_.

**Step 3:** Choose a pixel from {*P*_1_, *P*_2_, …, *P_M_*_×*N*-2_} and put it in the position of *P_M_*_×*N*-2_.

…

**Step *M×N*–1:** Choose a pixel from {*P*_1_, *P*_2_} and put it in the position of *P*_2_.

**Figure 3:**
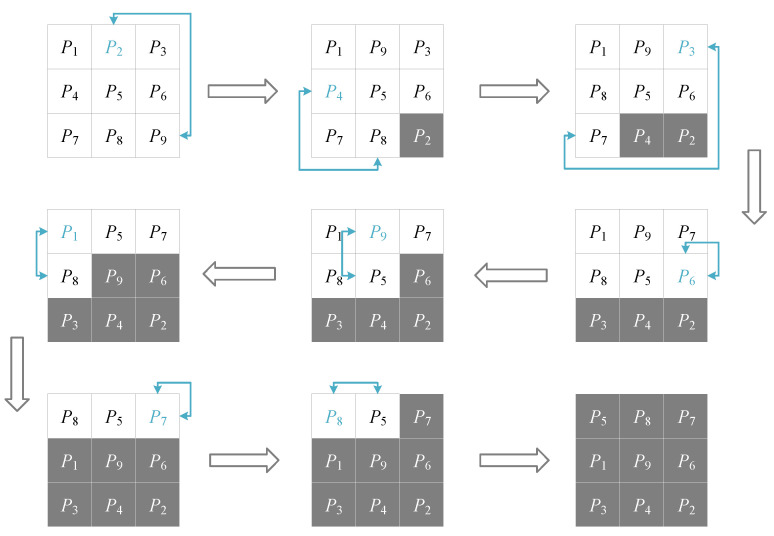
Process of generating a Hamiltonian path in an image of size 3 × 3. The blue pixels are chosen, and the grey pixels have been added into the path.

**Figure 4:**
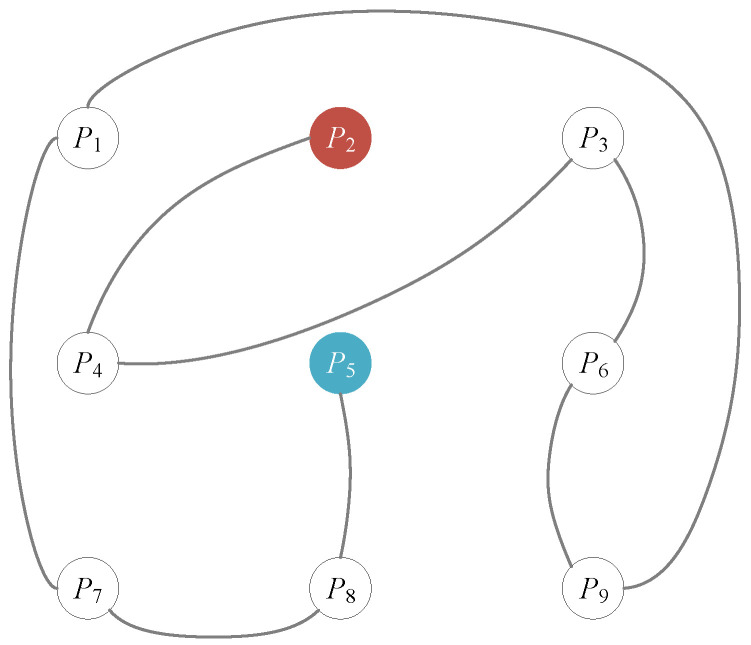
Generated Hamiltonian path. The red pixel is the beginning of the path and the blue pixel is the rear of the path.

In the above process, pixels that have been added into the path are insulated at the back of image’s pixel array. Among the whole image, these pixels are deemed as permutated pixels. The complete process is shown in Figure 3. Figure 4 illustrates the generated Hamiltonian path from a graph perspective.

### 2.3. Hamiltonian Path across Bit Planes

**Figure 5:**
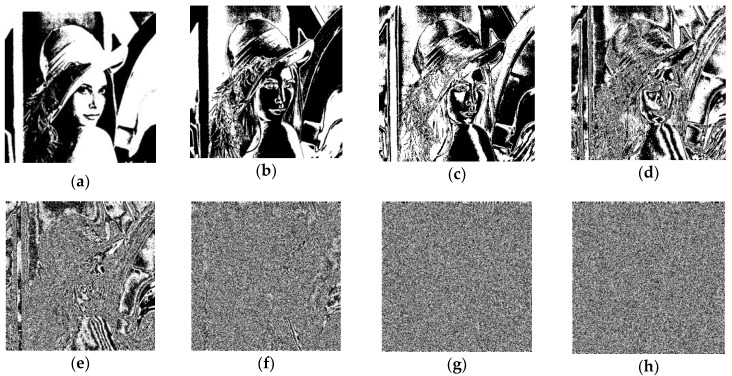
Bit planes of Lena, (**a**–**h**) are from the 8th bit plane to the 1st bit plane, respectively.

In [7], the intrinsic features of bit distribution in digital images were revealed. Higher bits of pixels hold higher weight of an image’s information, and there are strong correlations among the higher bit planes. In the instance of Figure 5, the 8th bit plane and the 7th bit plane tend to have opposite values. These features shall not be neglected in a secure cryptosystem.

**Figure 6:**
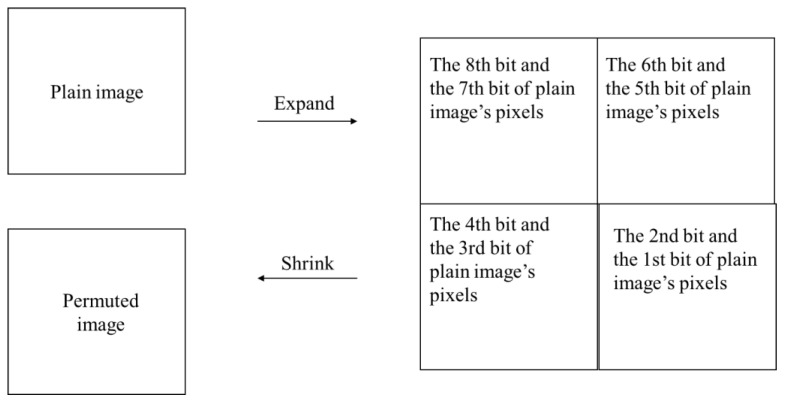
Modified expand–shrink strategy.

To build a Hamiltonian path across bit planes, the strategy of [7] was extended to greyscale images in this paper. By these means, a plain image of size *M* × *N* is expanded to 2*M* × 2*N*. All the bit planes of a plain image’s pixels were placed to the 1st bit plane and the 2nd bit plane of the expanded image’s pixels. After generating a Hamiltonian path, the bit planes were restored, and a permutated image of size *M* × *N* was formed. The whole procedure can be generalized into Figure 6.

**Figure 7:**
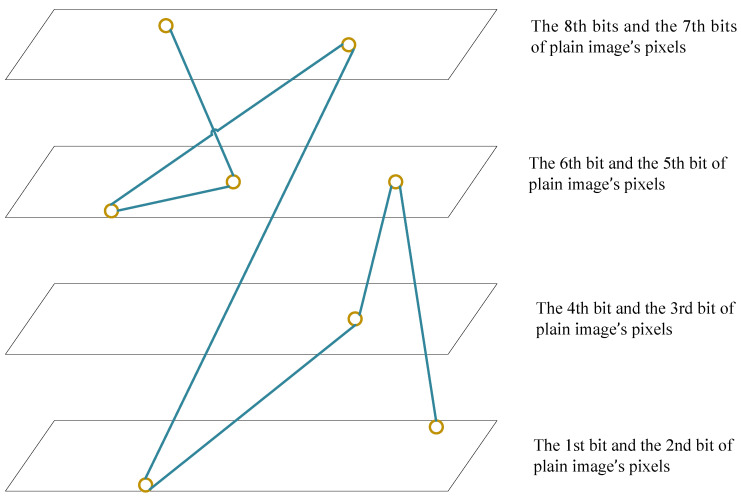
Hamiltonian path across bit planes.

Figure 7 is the illustration of the generated Hamiltonian path across bit planes.

## 3. Adjusted Bernoulli Map

To ensure the randomness of the Hamiltonian path, chaotic maps can serve as pseudo random number generators. Theoretically, any 1D chaotic map is compatible. In this section, an adjusted Bernoulli map is proposed.

### 3.1. Bernoulli Map

The original definition of Bernoulli map [22,23] is given by:

(1) $$x_{n+1}=2x_{n}\mathrm{mod}1=\left\{ \begin{array}{ll} 2x_{n} & 0<x_{n}<0.5\\ 2x_{n}-1 & 0.5\leq x_{n}<1 \end{array}\right..$$

**Figure 8:**
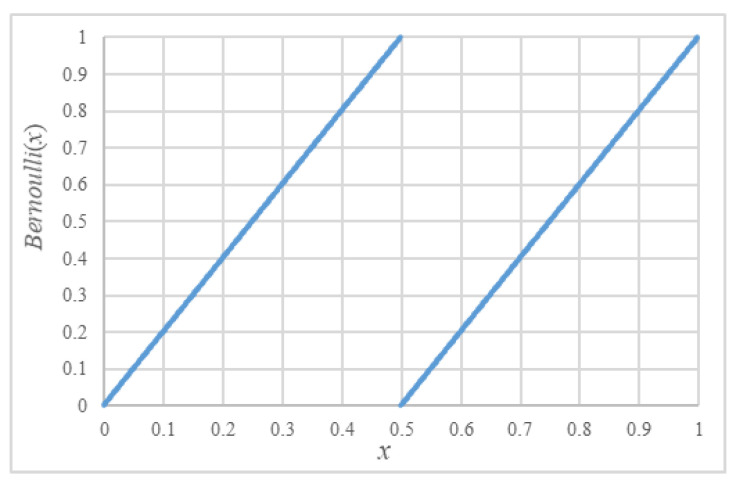
Bernoulli map.

The piecewise linear property of a Bernoulli map is demonstrated in Figure 8. When implemented into discrete computer systems, the map resembles bit shifting of floating numbers. Such degradation means that the original Bernoulli map is seldom applied to encryption algorithms directly.

### 3.2. Adjusted Bernoulli Map

To amplify the limited nonlinear property of original Bernoulli map, cascaded modulus operations are adopted in the adjusted Bernoulli map (ABM).

(2) $$x_{n+1}=\beta (\alpha x_{n}\mathrm{mod}1)\mathrm{mod}1.$$

**Figure 9:**
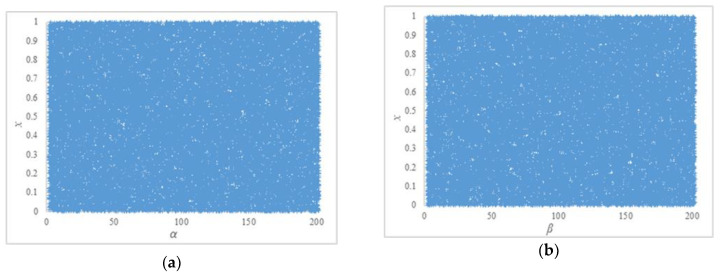
Bifurcation diagrams of ABM. (**a**) *β* = 3. The value of *α* is increased by 0.1, ranging from 2.1 to 202.1. (**b**) *α* = 3. The value of *β* is increased by 0.1, ranging from 2.1 to 202.1.

The parameters *α* and *β* can be many of the floating-point numbers that are bigger than two. Though the multiplication operation is linear in mathematics, the multiplication operation in computer systems involves the conversion between decimal number and binary number. The ABM possesses fair chaotic behavior in practice, especially when the parameters *α* and *β* are random. Owing to the finite precision of computers, the ABM does not work well when its parameters are big numbers, and special values such as 2**^N^** and 10**^N^** should be avoided. Here the **N** is the set of natural numbers. Part of the parameters’ value range is shown in Figure 9.

To examine the randomness of the pseudo-random numbers generated by the ABM, the NIST SP800-22 test suite [24] was utilized. In our experiment, 300 bitstreams of length 10^6^ were generated and tested. The *α* = 10.45678 and *β* = 10.123 in these bitstreams. The initial value of *x* was increased by 0.0033, ranging from 0.001 to 0.991. The test results are listed in Table 1.

## 4. Proposed Scheme

After the progress of Section 2, a bit-level permutation was completed. To obtain fair diffusion properties, XOR operations were performed on pixels, and their grey values were substituted dynamically. The whole encryption scheme is detailed in this section.

### 4.1. Encryption Algorithm

**Figure 10:**
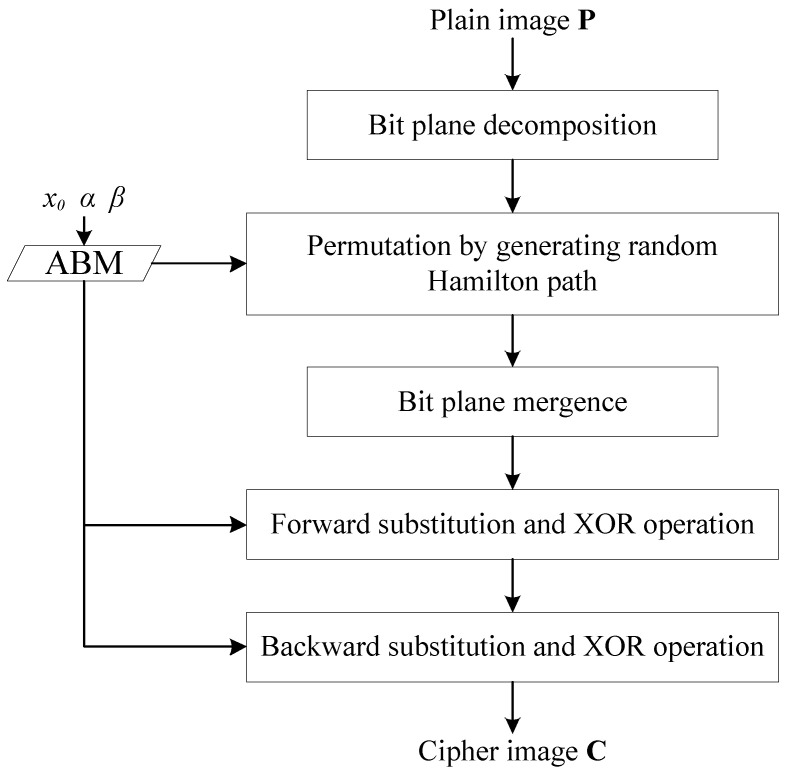
Encryption progress.

As is illustrated in Figure 10, the whole cryptosystem is handled by ABM. The inputs of the algorithm are the plain image *P* of size *M* × *N* and the parameters of ABM. The output is the cipher image *C*.

The whole encryption process is as below:

**Step 1:** Read in *P*. Iterate ABM to avoid transient effect.

**Step 2:** Decompose *P*’s bit planes. Make a montage of these bit planes to obtain an image *B* of size 2*M* × 2*N*.

**Step 3:** For *i* = 2*M* × 2*N*, 2*M* × 2*N* − 1, 2*M* × 2*N* − 2, …, 3, 2, iterate ABM to generate pseudo random number *r_i_* and use Equation (3) to quantize it. Swap *M*’s *i*th pixel *M_i_* and *j*th pixel *M_j_*.

(3) $$j=round(r_{i}\times 10^{14})\mathrm{mod}i+1.$$

**Step 4:** Merge the decomposed bit planes to obtain the permutated image *H* of size *M* × *N*.

**Step 5:** Initialize two 1D arrays or vectors *S* and *T* by Equation (4). For *i* = 0, 1, 2, …, 255,

(4) $$S_{i}=T_{i}=i.$$

**Step 6:** For *i* = 255, 254, …, 2, 1, iterate ABM to generate pseudo random number *u_i_* and use Equation (5) to quantize it. Swap *S_i_* and *S_j_*.

(5) $$j=round(u_{i}\times 10^{14})\mathrm{mod}i.$$

**Step 7:** For *i* = 255, 254, …, 2, 1, iterate ABM to generate pseudo random number *p_i_* and use Equation (6) to quantize it. Swap *T_i_* and *T_j_*.

(6) $$j=round(p_{i}\times 10^{14})\mathrm{mod}i.$$

**Step 8:** For *i* = 1, 2, …, *M* × *N* − 1, use Equation (7) to diffuse *H*’s pixel *H_i_*_+1_. Here, *a_i_* is the pseudo random number generated by ABM.

(7) $$H_{i+1}=H_{i+1}⊕S_{T_{H_{i}}}⊕(round(a_{i}\times 10^{14})\mathrm{mod}256).$$

**Step 9:** For *i* = *M* × *N*, *M* × *N* − 1, …, 3, 2, use Equation (8) to diffuse *H*’s pixel *H_i_*_−1_. Here, *b_i_* is the pseudo random number generated by ABM.

(8) $$H_{i-1}=H_{i-1}⊕T_{S_{H_{i}}}⊕(round(b_{i}\times 10^{14})\mathrm{mod}256).$$

**Step 10:** Save *H* as the *C*.

### 4.2. Discussion

In Step 2, Step 3, and Step 4, the permutation phase that works on a bit-level was performed. According to the method of building a Hamiltonian path, two arrays were generated for grey value’s substitution in Step 5, 6, and 7. The arrays were arrangements of integers from 0 to 255, in accordance with pixel’s grey levels. As the arrays were randomly generated, there were 256! ≈ 8.578 × 10^506^ possible arrangements. In this way, the modification of plain images could be amplified and transmitted in Step 8 and Step 9, causing an avalanche effect.

### 4.3. Decryption Algorithm

**Figure 11:**
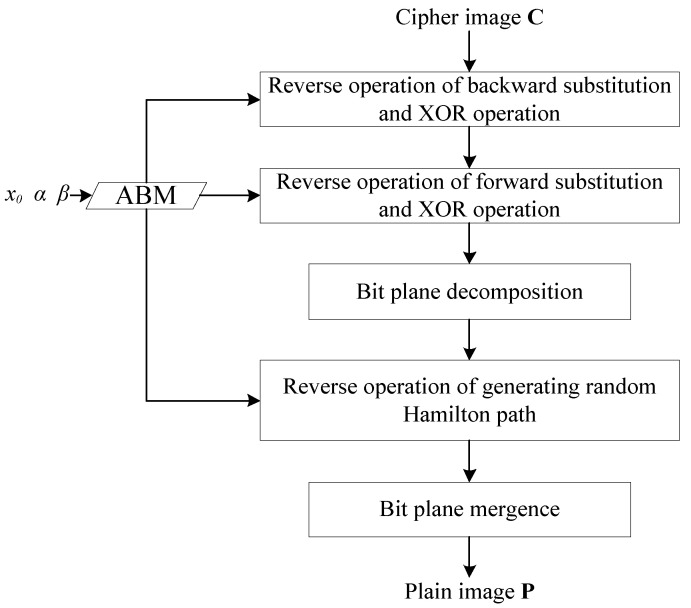
Decryption progress.

The decryption algorithm is the reverse progress of the encryption algorithm, as can be seen from Figure 11.

In the encryption process, the substitution is realized by arrays *S* and *T*. The reverse operation of substitution needs the inverse map of *S* and *T*, which can be generated by Equation (9).

(9) $$S_{S}^{\prime }{}_{{}_{i}}=T_{T_{i}}^{\prime }=i.$$

In the formula, *i* = 255, 254, …, 2, 1, 0. The *S’* and *T’* are the inverse maps of *S* and *T,* respectively.

## 5. Simulation Experiments

To check the performance of the proposed scheme, the results of simulation experiments were evaluated by several criteria in this section. Our experimental environment was a desktop PC with 64-bit Windows 10 OS, Intel i7-2600 CPU, and 8GB RAM. The programming language was C++, and the developing environments were Visual Studio 2019 and OpenCV 4.1.0. The test images were chosen from SIPI image database [25].

### 5.1. Secret Key Analysis

**Figure 12:**
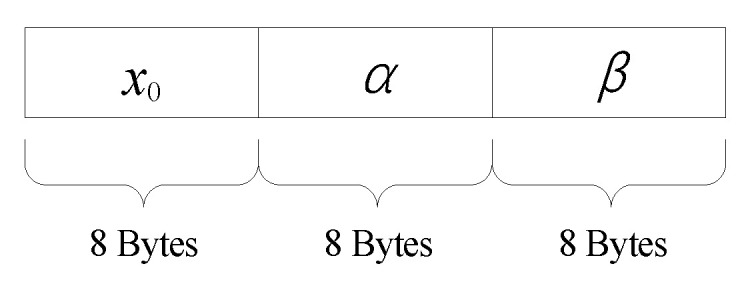
Encryption process.

Secret key is an indispensable component of a cryptosystem. The key space is suggested to be no less than 2^100^ [26]. The secret keys of the proposed scheme are parameters of ABM. In our simulation experiment, the data type of the keys was double precision floating point numbers. According to IEEE 754 standard, each key occupies 8 Bytes and owns significant digit of 52 bits. The structure of secret key is as shown in Figure 12, and the key space is bigger than 2^100^.

To examine the key sensitivity in the encryption process and decryption process, a strict test for bit change rate—NBCR (the number of bit change rate) [27]—was utilized:

(10) $$NBCR(C_{1},C_{2})=\frac{Ham(C_{1},C_{2})}{M\times N\times d}$$

In the above formula, *C*_1_ and *C*_2_ are two images of size *M* × *N* and bit-depth d. *Ham*(*C*_1_,*C*_2_) represents the Hamming distance between *C*_1_ and *C*_2_; in other words, the number of different bits between the two images. The calculation results of NBCR should be close to 50%, which indicates that around 50% of the bits are different between *C*_1_ and *C*_2_.

In our work, three groups of modified keys were set as the illegal keys. These illegal keys are utilized to encrypt the plain images in the encryption process and decrypt the cipher images in the decryption process. The obtained encrypted images and decrypted images were made in comparison with the original plain images and cipher images. The NBCRs are listed in Table 2.

### 5.2. Histograms

The histogram is the foundation of various spatial image processing techniques, e.g., image enhancement. Moreover, the inherent information of histograms is useful in image compression and segmentation. For an image of size *M* × *N* and bit-depth *d*, the histogram is a discrete function:

(11) $$h(r_{i})=q_{i}.$$

Here *i* = 0, 1, 2, …, 2*^d^* − 1, *q_i_* is the pixels’ quantity of grey value *r_i_*. The variance of histogram can be calculated by Equation (12).

(12) $$Var(h_{i})=\frac{1}{2^{d}}\sum_{i=0}^{2^{d}-1}{\left(q_{i}-\mu_{h}\right)}^{2}.$$

The $\mu_{h}$ is the arithmetic mean value of *q_i_*. The histogram of a cipher image should be relatively uniform. After encryption, the variance of an image’s histogram should be reduced. In Table 3, the variance of several images’ histograms are listed.

In practice, histograms are often normalized by Equation (13).

(13) $$p(r_{i})=\frac{q_{i}}{M\times N}.$$

**Figure 13:**
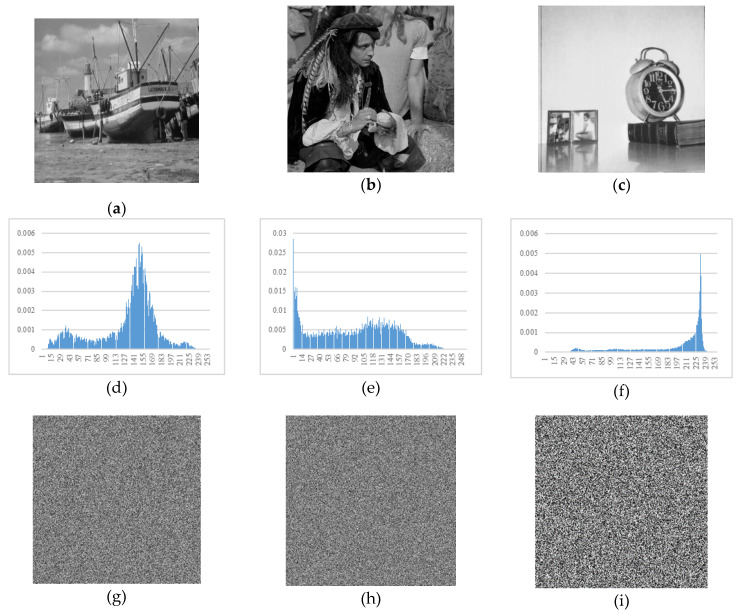

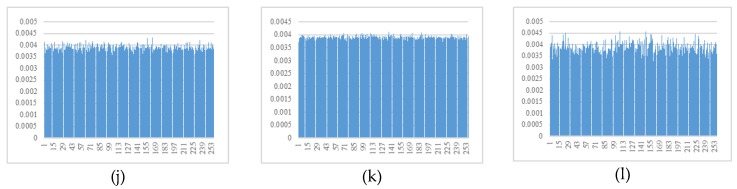
Histograms. (**a**) Plain image boat; (**b**) plain image male; (**c**) plain image clock; (**d**) histogram of plaintext boat; (**e**) histogram of plaintext male; (**f**) histogram of plaintext clock; (**g**) cipher image boat; (**h**) cipher image male; (**i**) cipher image clock; (**j**) histogram of cyphertext boat; (**k**) histogram of cyphertext male; (**l**) histogram of cyphertext clock.

After normalization, *p*(*r_i_*) represents the emergence probability of the *i*th grey value. The normalized histograms of plain images and cipher images are shown in Figure 13.

### 5.3. Information Entropy

Information entropy was proposed by C. E. Shannon, which is a measurement of information’s randomness. For a digital image, it is hard to predict the content if its information entropy is high. The calculation formula of information entropy is as shown in Equation (14). Here, the *p*(*r_i_*) is identical to Equation (13).

(14) $$H=-\sum_{i=0}^{2^{d}-1}p(r_{i})\log_{2}p(r_{i}).$$

The ideal value of a cipher image’s information entropy is its bit-depth *d*. In Table 4, the information entropy of plain images and cipher images are listed.

### 5.4. Differential Attack

To resist differential attacks, tiny modification in plain images should cause massive changes in the cipher image. This is known as diffusion property in cryptography. NPCR (number of pixel change rate) and UACI (unified averaged changed intensity) are two common indicators for an algorithm’s ability of resisting differential attacks [29]. If *C*_1_ and *C*_2_ are two images of size *M* × *N* and bit-depth *d*, then

(15) $$NPCR=\left[\frac{1}{M\times N}\sum_{i=1}^{M}\sum_{j=1}^{N}D(i,j)\right]\times 100\%$$

(16) $$UACI=\left[\frac{1}{M\times N\times 255}\sum_{i=1}^{M}\sum_{j=1}^{N}\left|C_{1}(i,j)-C_{2}(i,j)\right|\right]\times 100\%.$$

Here,

(17) $$D(i,j)=\left\{ \begin{array}{l} 0,C_{1}(i,j)=C_{2}(i,j)\\ 1,C_{1}(i,j)\neq C_{2}(i,j) \end{array}\right..$$

In our experiment, the plain image boat of size 512 × 512 was utilized for evaluating the diffusion effect. Some pixels were chosen in the image, and the last bit of these pixels were reversed, respectively. Then, the modified images were encrypted. As can be seen from Table 5, the NPCRs and UACIs were close to theoretical values after two encryption rounds.

### 5.5. Correlation Coefficients

Plain images usually are redundant in the spatial domain, which means that adjacent pixels are highly correlated. Whereas, in cipher images, such a correlation should be broken. To measure the correlation between adjacent pixels, we calculated correlation coefficients as below:

(18) $$r_{xy}=\frac{E[(x-\mu_{x})(y-\mu_{y})]}{\sigma_{x}\sigma_{y}}.$$

The *x* and *y* are pixel vectors of the same length. The *μ_x_* and *μ_y_* are their arithmetic mean values, and the *σ_x_* and *σ_y_* are their standard deviations. The range of correlation coefficients is [−1, 1]. If *x* and *y* are not correlated, *r_xy_* shall be close to 0.

**Figure 14:**
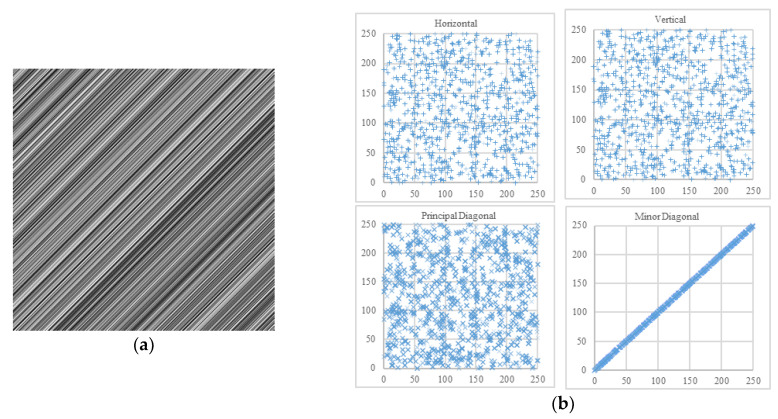
One example. (**a**) An image in which all adjacent pixels of minor diagonal direction are equal; (**b**) its scatter plots.

**Figure 15:**
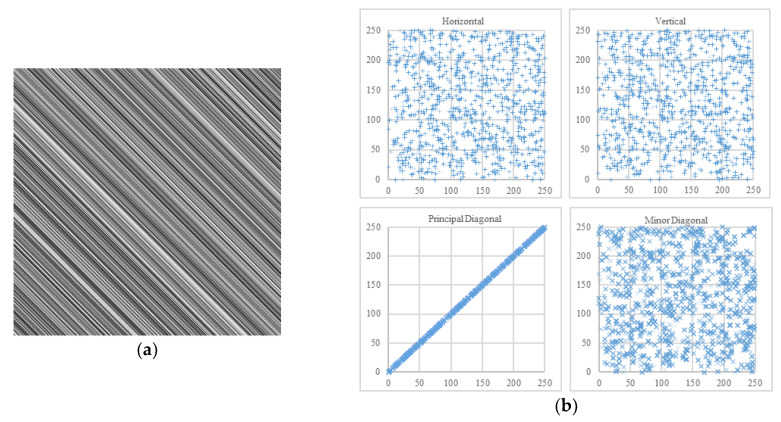
Another example. (**a**) An image in which all adjacent pixels of principal diagonal direction are equal; (**b**) its scatter plots.

Commonly, the adjacent pixels of three directions are calculated, respectively horizontal, vertical, and diagonal. Whereas, there exist two orthogonal diagonal directions in 2D matrices of pixels—the principal diagonal direction (from upper-left to lower-right) and the minor diagonal direction (from upper-right to lower-left). For instance, in a pixel block $\left[\begin{array}{cc} p_{1} & p_{2}\\ p_{3} & p_{4} \end{array}\right]$, *p*_1_ and *p*_4_ are adjacent in the principal diagonal direction, while *p*_2_ and *p*_3_ are adjacent in the minor diagonal direction. In the field of image encryption, the definition of diagonal direction is usually ambiguous. However, the two diagonal directions are nonequivalent for some image processing techniques and image encryption algorithms [30,31,32,33,34]. Under the extreme circumstances in Figure 14 and Figure 15, it is not enough to calculate only three of the four directions.

For all the plain images and cipher images in our experiments, correlation coefficients of 10,000 adjacent pixel pairs in each of the four directions were calculated. The results are listed in Table 6.

### 5.6. Efficiency

In the proposed scheme, bit-level permutation is performed in linear time complexity. Meanwhile, the diffusion phase is also linear. If the encrypted image is of size *M* × *N*, the algorithm’s time complexity is *O*(*MN*). The time complexity of the algorithm in [4] is also *O*(*MN*). However, our bit-level scheme is slower than the pixel-level scheme of [4]. In [2], permutation was companied by sorting operation. Thus, the scheme’s efficiency was related to the adopted sorting algorithm. In [28], the algorithm’s time complexity is *O*(*MN*(*M* + *N*)). The comparison between these algorithms’ efficiency is presented in Table 7.

## 6. Conclusions

In this paper, a 1D adjusted Bernoulli map is proposed, which is suitable for encryption systems. Based on the new chaotic map, an innovative image encryption algorithm was designed. The permutation phase was realized by generating a random Hamiltonian path, which was performed across different bit planes. Then, the idea of random Hamiltonian path was extended for substitution of grey levels in the diffusion phase. Various criterions indicate that our scheme had a pretty good performance. Besides, for measuring the correlation of adjacent pixels more reasonably, both the principal diagonal direction and the minor diagonal direction are involved when calculating correlation coefficients.

## Figures and Tables

**Table 1:** Randomness test using NIST SP800-22 test suite.

Statistical Tests	P-value	Pass Rate (%)
Frequency	0.798139	100.00
Block frequency	0.108791	99.33
Cumulative Sums *	0.282804	99.83
Runs	0.588652	99.67
Longest run	0.245072	99.33
Rank	0.319084	100.00
FFT	0.280306	99.00
Non overlapping template *	0.468139	98.95
Overlapping template	0.425059	98.00
Universal	0.449672	99.33
Approximate entropy	0.561227	99.67
Random excursions *	0.533005	98.95
Random excursions variant *	0.419542	99.27
Serial *	0.464632	98.83
Linear complexity	0.915745	99.33

∗ Average value of multiple tests.

**Table 2:** Key sensitivity (Δ = 0.00000000000001).

		Boat (512 × 512)	Couple (512 × 512)	Tank (512 × 512)	Male (1024 × 1024)	Clock (256 × 256)
Key sensitivity in encryption process	(*x*_0_ + Δ, *α*, *β*)	0.499321	0.499997	0.500057	0.499904	0.501156
	(*x*_0_, *α* + Δ, *β*)	0.499923	0.500155	0.499765	0.499937	0.500164
	(*x*_0_, *α*, *β* + Δ)	0.49994	0.500076	0.500499	0.500078	0.500856
Key sensitivity in decryption process	(*x*_0_ + Δ, *α*, *β*)	0.500289	0.499741	0.500082	0.499858	0.500328
	(*x*_0_, *α* + Δ, *β*)	0.500154	0.499415	0.5001	0.49992	0.501308
	(*x*_0_, *α*, *β* + Δ)	0.499747	0.500337	0.499742	0.49986	0.499378

**Table 3:** Variance of histograms.

	Plain Image	Cipher Image
Chemical plant (256 × 256)	50,326.4	248.469
Clock (256 × 256)	282,062	248.328
Moon surface (256 × 256)	135,688	248.094
Boat (512 × 512)	1,535,880	1137.66
Couple (512 × 512)	1,195,460	1002.11
Lena (512 × 512)	632,254	986.281
Tank (512 × 512)	8,103,600	1043.73
Airplane (1024 × 1024)	115,199,000	3783.7
Airport (1024 × 1024)	31,596,400	3832.03
Male (1024 × 1024)	11,349,400	4412.8

**Table 4:** Information entropy.

	Plain Image	Proposed Scheme	[2]	[4]	[28]
Chemical plant(256 × 256)	7.34243	7.99725	7.99716	7.99692	7.99683
Clock(256 × 256)	6.70567	7.99727	7.99726	7.99692	7.99705
Moon surface(256 × 256)	6.70931	7.99725	7.99738	7.9974	7.9972
Boat(512 × 512)	7.19137	7.99922	7.99934	7.9994	7.99921
Couple(512 × 512)	7.20101	7.99931	7.99934	7.99931	7.99936
Lena(512 × 512)	7.44551	7.99932	7.99929	7.99934	7.99932
Tank(512 × 512)	5.49574	7.99928	7.99934	7.99923	7.99934
Airplane(1024 × 1024)	5.64145	7.99984	7.99984	7.99983	7.99981
Airport(1024 × 1024)	6.83033	7.99984	7.99983	7.99981	7.99983
Male(1024 × 1024)	7.52374	7.99981	7.99978	7.99981	7.99981

**Table 5:** Results of NPCR and UACI.

Index of Modified Pixel	NPCR (1 Round)	UACI (1 Round)	NPCR (2 Rounds)	UACI (2 Rounds)
0	0.996983	0.3349	0.995941	0.335169
255	0.99733	0.335224	0.996063	0.3338
511	0.996616	0.334727	0.996143	0.333902
65,151	0.99897	0.3355	0.996078	0.333911
65,407	0.998333	0.335641	0.996254	0.334896
130,560	0.996365	0.333719	0.995861	0.334763
130,816	0.99995	0.335614	0.996147	0.334734
131,071	0.999985	0.335876	0.996216	0.335797
196,096	0.999943	0.336146	0.995804	0.333875
196,352	0.999031	0.335429	0.996269	0.334645
261,632	0.9981	0.335204	0.996181	0.333829
261,888	0.999249	0.335551	0.996037	0.334519
262,143	0.997608	0.335361	0.995998	0.334605
**Theoretical value**	0.996094	0.334635	0.996094	0.334635

**Table 6:** Correlation coefficients.

		Boat	Male	Clock	Figure 14a	Figure 15a
Plain image	Horizontal	0.936502	0.978016	0.956658	−0.0104305	−0.0347679
	Vertical	0.970165	0.981711	0.973594	−0.0262352	−0.0253942
	Principal diagonal	0.922103	0.965681	0.940988	0.0309025	1
	Minor diagonal	0.924285	0.967724	0.934225	1	0.00261237
Proposed scheme	Horizontal	−0.00790818	−0.00530914	−0.00627326	0.0106215	0.00724055
	Vertical	−0.0032019	−0.00593338	−0.00787923	0.0000261687	0.00380631
	Principal diagonal	−0.00753223	0.0180877	−0.00519652	0.000707789	−0.0161754
	Minor diagonal	0.001262	0.00994515	−0.00729377	−0.0060987	0.00262413
[2]	Horizontal	0.0272732	0.00444088	0.0105537	0.00498476	−0.00189389
	Vertical	−0.0321433	−0.000856047	−0.00733777	−0.0126175	0.0129397
	Principal diagonal	−0.00603878	−0.00964336	−0.0138118	0.0118652	−0.0067004
	Minor diagonal	−0.0013256	0.0046903	−0.00501911	0.00299615	−0.0185178
[4]	Horizontal	0.00361182	−0.00595886	−0.00236848	0.000340202	−0.0171794
	Vertical	0.00145023	−0.0103426	−0.00437046	0.00520304	0.00879099
	Principal diagonal	0.00395435	0.00305054	−0.000705693	−0.0120762	−0.00874923
	Minor diagonal	−0.000165327	0.00232492	0.000369637	−0.00743531	−0.00299425
[28]	Horizontal	0.00899491	0.00754775	0.000411031	0.0057195	−.00967912
	Vertical	−0.0041634	0.000629605	−0.00538419	−0.00266845	0.0105734
	Principal diagonal	−0.00463651	0.00000710876	0.011115	−0.0034216	−0.00922371
	Minor diagonal	0.0127711	0.00677395	0.00671256	0.0121195	0.00781511

**Table 7:** Efficiency of algorithms.

	Proposed Tcheme	[2]	[4]	[28]
Chemical plant(256 × 256)	0.008 s	0.02 s	0.004 s	0.03 s
Clock(256 × 256)	0.008 s	0.019 s	0.003 s	0.028 s
Moon surface(256 × 256)	0.008 s	0.019 s	0.003 s	0.029 s
Boat(512 × 512)	0.029 s	0.093 s	0.011 s	0.236 s
Couple(512 × 512)	0.03 s	0.094 s	0.012 s	0.234 s
Lena(512 ×512)	0.028 s	0.09 1s	0.009 s	0.239 s
Tank(512 × 512)	0.029 s	0.091 s	0.011 s	0.243 s
Airplane(1024 × 1024)	0.129 s	0.441 s	0.032 s	2.392 s
Airport(1024 × 1024)	0.121 s	0.44 7s	0.033 s	2.388 s
Male(1024 × 1024)	0.116 s	0.434 s	0.033 s	2.398 s
**Average throughput**	66.062 Mbps	21.884 Mbps	194.571 Mbps	9.542 Mbps
